# All you need is love: preliminary validation of a new test of 10 parenting competencies that predict good outcomes with young children

**DOI:** 10.3389/fpsyg.2026.1796786

**Published:** 2026-08-10

**Authors:** Robert Epstein, Priyanka Nanayakkara, Amanda Newland, Ning Wang, Vanessa R. Zankich

**Affiliations:** American Institute for Behavioral Research and Technology, Vista, CA, United States

**Keywords:** EPCI, Epstein Parenting Competencies Inventory, parenting competencies, parenting outcomes, parenting skills, test validation

## Abstract

**Objectives:**

The main objectives of this study were to provide preliminary evidence of the validity of a new online test of parenting competencies and to determine the relative value of 10 competencies that have been shown in empirical studies to be helpful in parenting.

**Methods:**

We examined data obtained from a diverse convenience sample of 16,986 parents in 133 countries (53.3% from the U.S.) who completed the new online parenting questionnaire. We employed a concurrent study design to assess the validity of the new instrument. In a double-blind procedure to assess content validity, 11 parenting professionals rated both the competencies and the questionnaire items. To assess criterion validity, we used regression analyses to examine the relationship between questionnaire scores and participants’ answers to questions about the quality of their relationship with their children, how highly they rated their own parenting ability, and the happiness, health, and success of their children.

**Results:**

Total scores were positively correlated with answers to our five criterion questions (*p* < 0.001), suggesting relatively strong criterion validity for the new questionnaire. Internal consistency reliability was also high. Regression analyses suggested that the competency we labeled Love and Affection was the best predictor of good outcomes with children and that the competency we called Safety was the least valuable of the 10. Effects were found for gender (with females slightly outscoring males), race/ethnicity, sexual orientation, age, number of children, and education level. Total scores were higher for parents who had had parenting training, and more training was associated with higher scores.

**Conclusion:**

The study supports the view that parenting can be broken down into measurable and trainable competencies and provides guidance regarding how such training should be conducted.

## 1 Introduction

The objectives of the present study were to (a) introduce and evaluate the Epstein Parenting Competencies Inventory (EPCI)—a new instrument the authors developed for assessing 10 behavioral competencies that past empirical research has shown to be associated with effective parenting, (b) analyze data from a large, international group of people who completed the English version of the EPCI online, (c) compare the relative value of the 10 competencies, and (d) analyze the data from a demographic perspective. By using a competencies approach, we provide both the public and practitioners with a practical tool for measuring and potentially improving parenting practices and outcomes.

A competencies approach to parenting is, we believe, consistent with the desire most parents have to be the best parents they can be. At this writing, Amazon.com lists more than 60,000 books on dieting but more than 70,000 books on how to parent. There are even books that do nothing but compare various approaches to parenting (e.g., Cohen, 2002; Gross-Loh, 2014; Hoefle, 2012). Unfortunately, relatively few parents rely on empirical studies to guide their parenting practices (Bornstein et al., 2010). Instead, most parents simply parent the way their own parents did (Civitas Initiative et al., 2000; Radey and Randolph, 2009; Sistler and Gottfried, 1990), or, in some cases, if they had had difficult childhoods, parent in ways they perceive as opposite to the practices of their parents (Kaufman and Zigler, 1993). Besides firsthand experiences, parents also rely on proximal social networks such as families and friends (Belsky et al., 1989; Civitas Initiative et al., 2000; Smith et al., 1994; Sparling and Lowman, 1983) before consulting external sources such as educational materials and professionals (Geboy, 1981).

Meanwhile, since research on parenting styles was first introduced in the 1960s (Baumrind, 1967, 1971; cf. Maccoby and Martin, 1983), an increasing body of empirical evidence has identified particular parenting styles that are associated with a variety of positive outcomes: better relationships between parents and their children (Cox and Harter, 2003; Steele and McKinney, 2019), healthier children (Arredondo et al., 2006; Fogelholm, 1999; Iotti et al., 2023), better socio-emotional development (Davidov and Grusec, 2006; Li et al., 2023), higher academic achievement (Baumrind et al., 2010; Malahat et al., 2019), and higher likeliness to engage in prosocial behavior (Iotti et al., 2023).

### 1.1 Instruments that assess parenting

Over the years, a number of instruments have been developed to measure various aspects of parenting styles, some inspired by the pioneering work of Diana Baumrind and her colleagues. Robinson et al. (1995) developed the 62-item Parenting Practices Questionnaire that measures parenting styles based on Baumrind’s three dimensions of parenting styles: authoritarian, authoritative, and permissive. Robinson et al. (1995) combined 80 items from Block’s (1965) Child-Rearing Practices Report with 53 newly-constructed items based on Baumrind’s work. One thousand two hundred and fifty-one parents of preschool and school-age children were given the 133-item questionnaire, the results of which were reduced using a factor analysis. A final count of 62 items were then categorized into factors corresponding to authoritarian, authoritative, and permissive parenting, and significant correlations were found between parenting styles and desirable outcomes. According to the contextual model of parenting style and practices proposed by Darling and Steinberg (1993), parenting style “influences child development primarily through its moderating influence on the relationship between parenting practices and developmental outcomes” (p. 493). This model suggests that parenting style is predictive of specific practices that influence child development (cf. Abidin et al., 2022; Neel et al., 2018). The Revised Parents as a Social Context Questionnaire (Egeli et al., 2015), which identifies six attributes of parenting styles (Warmth, Rejection, Structure, Chaos, Autonomy Support, and Coercion), has also been used to study parenting outcomes.

Instruments also exist that look at parenting attitudes, such as the Parental Attitudes Toward Childrearing Questionnaire (Easterbrooks and Goldberg, 1984) and the Parenting Questionnaire (Budd et al., 2012, 2015). According to Holden and Edwards (1989), parental attitudes predict parenting practices, which are an important part of a child’s environment (cf. Francis and Filmore, 1934; Updegraff, 1939) and which play an important role in shaping a child’s behavior and personality (Symonds, 1949). Winder and Rau (1962) suggested that parental attitudes such as ambivalence, punitiveness, restrictiveness, and low maternal self-esteem are correlated with social deviance in boys.

Other instruments look more directly at parenting practices. The Alabama Parenting Questionnaire (Dadds et al., 2003) measures degree of interaction, positive parenting, degree of supervision, consistency of punishment, and the use of corporal punishment to predict the conduct of children. The Comprehensive General Parenting Questionnaire (Sleddens et al., 2014) assesses five main parenting constructs: Nurturance, Overprotection, Coercive Control, Behavioral Control, and Structure. The Mindfulness in Parenting Questionnaire (McCaffrey et al., 2016) measures mindful parenting practices that might improve the parent-child relationship through increased trust and decreased stress (cf. McKee et al., 2018).

Tests also exist that measure various aspects of parenting competence. The Parenting Sense of Competence Scale assesses (as one might expect) a parent’s sense of competence (Ohan et al., 2000; Rogers and Matthews, 2004). One project of special note that measured parenting competencies directly originated at Kansas State University (KSU) and was funded by the U.S. Department of Agriculture (USDA) and KSU. A panel of four professors worked together to develop an evidence-based list of 29 critical parenting practices organized into six themes: (1) Care for Self, (2) Understand, (3) Guide, (4) Nurture, (5), Motivate, and (6) Advocate (Smith et al., 1994). Eighty-nine parenting specialists who were part of the USDA’s Cooperative Extension System—a non-credit educational system for farmers, ranchers, and others that the USDA has supported for more than a century—were then asked to weigh in on the parenting education model the panel had developed. The resulting National Extension Parenting Education Model (NEPEM) was thus developed by consensus, according to the authors. The model was summarized in a 88-page report published by the KSU’s Cooperative Education Service in 1994 (Smith et al., 1994), and a condensed version of that report was also published that year in a scholarly journal (Goddard et al., 1994). Shortly after, one of the members of the original panel, H. W. Goddard, and others published a brief paper describing a questionnaire—the Parent Self-Evaluation Instrument—that parents could use to rate how well their parenting practices matched those recommended by NEPEM (Edgmon et al., 1996).

We are not aware of studies designed to validate NEPEM. It was developed, however, by experts who relied on relevant studies available at the time, and thus the model has an empirical basis. The NEPEM-related questionnaire was never validated or widely adopted, as far as we are aware, but NEPEM itself has been used as a guide for developing parent education programs, and the effectiveness of some of those programs has been reported (Behrendt, 2009; Debord et al., 2006; Goddard and Marshall, 2006; Guin et al., 2018). NEPEM is relevant to the present study because it catalogs 29 parenting practices (sometimes referred to as “skills”) said to be helpful in producing positive parenting outcomes. Although NEPEM does not speak of competencies *per se*, the skill sets it describes overlap with the competencies that are evaluated and ranked in the present study. We summarize the overlap in Table 1. Thirteen of the 29 practices in NEPEM are well represented in the questionnaire that is the subject of the present study. Sixteen NEPEM practices are not represented (see endnote, Table 1). Depending on how one chooses to count them, our questionnaire contains between 58 and 75 items that are not represented in NEPEM, mainly because our questionnaire items pinpoint specific behaviors rather than make broad recommendations.

**TABLE 1:** Comparison of the Epstein Parenting Competencies Inventory (EPCI) competencies and national extension parenting education model (NEPEM) practices.

EPCI competencies	EPCI scored items	Corresponding NEPEM practices^†^
1. Autonomy and independence	I generally support my child’s decisions.	Provide children with developmentally appropriate opportunities to learn responsibility.
	I regularly give my child chores to do in order to teach him or her how to handle responsibility.	
	I try to teach my child how to solve problems on his or her own.	Teach problem-solving skills.
	I try to encourage my child to express his or her individuality and uniqueness.	
	I always treat my child with respect.	Foster children’s self-respect and hope.
	I encourage my child to set goals for him- or herself.	Provide children with developmentally appropriate opportunities to learn responsibility.
	I try to teach my child how to manage his or her emotions.	
	I generally encourage my child to make his or her own choices.	
	I often ask for my child’s opinions.	
2. Behavior management	I reward my child far more often than I punish him or her.	
	When I penalize my child, I make sure that he or she has some way to earn back what was lost.	
	I continually try to lead by positive example so my child can imitate my good behavior.	Model appropriate desired behavior.
	I only punish my child for the purpose of teaching and never because I am out of control.	
	I never punish my child for something he or she did accidentally.	
	I often reward my child’s good behavior.	
	I regularly reward my child for following my instructions.	Establish and maintain reasonable limits.
	I try to reward my child’s good behavior immediately so my child can associate the reward with the good behavior.	
	I try to teach my child positive methods for managing the behavior of other.	
3. Education and learning	I encourage my child to get good grades.	
	I read and try to learn new things in order to serve as a good role model for my child.	Model appropriate desired behavior.
	I encourage my child to read.	
	I encourage my children to keep up with current events.	
	I regularly try to attend events at my child’s school.	
	I try to make sure that my child completes homework assignments on time.	
	I keep in touch with my child’s teachers.	
	I make sure my child always has the school supplies and educational resources he or she needs.	
	I try to be available to my child if he or she needs help with his or her homework.	
4. Healthy lifestyle	I encourage my child to bathe regularly, brush his or her teeth, and in other ways to maintain good hygiene.	
	I encourage my child to exercise regularly.	
	I try to make sure that my child gets restful and adequate sleep.	
	I try to involve my child in healthful outdoor activities.	
	I set a good example for my child by eating healthful foods	Model appropriate desired behavior.
	I regularly take my child to the doctor and dentist for check-ups.	
	I try to exercise regularly to serve as a good role model for my child.	Model appropriate desired behavior.
	I try to serve healthful meals at home.	
	I’ve warned my child about the dangers of illegal drugs and try to stay aware of his or her possible drug use.	
5. Life skills	I manage money well.	Manage family resources.
	I always provide adequate food, clothing, and shelter for my child.	Provide for the nutrition, shelter, clothing, health, and safety needs of one’s children.
	I occasionally struggle with serious bad habits, such as alcoholism, drug abuse, or gambling. (reversed scored)	
	I constantly strive to improve myself.	
	When I am employed, I always show up for work prepared and on time.	
	I typically have a steady income sufficient to cover my expenses.	
	I think about and plan for the future.	
	I am always prepared for emergencies and possible hard times.	
	I keep my home clean and well organized.	
6. Love and affection	I sometimes do nice things for my child just because I love him or her.	Express affection and compassion.
	I always try to be aware of my child’s hopes and dreams.	Listen and attend to children’s feelings and ideas.
	I never put down or insult my child.	
	I’ve been physically affectionate with my child over the years in ways suited to his or her age and needs.	Express affection and compassion.
	I’m always available when my child needs someone to talk to.	Listen and attend to children’s feelings and ideas.
	I always encourage my child to be positive and caring toward other people.	Teach kindness.
	I often reassure my child that when he or she does something wrong, I still love him or her.	Express affection and compassion.
	I always show my child that I’m listening carefully by expressing interest in what he or she is saying.	Listen and attend to children’s feelings and ideas.
	No matter how busy I am, I try to spend quality time with my child.	Express affection and compassion.
7. Relationship skills	I am careful never to say negative things to my child about other people who are important in his or her life.	
	I have always tried to teach my child to maintain positive and constructive relationships with other people.	
	When in a relationship, I always try to be aware of and to fulfill my partner’s needs.	
	When in a relationship, I try not to argue in front of my child.	
	Whenever it’s appropriate, I try to coordinate my parenting with the other parent.	Cooperate with one’s child-rearing partners.
	When in a relationship, I often have trouble communicating with my partner. (reversed scored)	Cooperate with one’s child-rearing partners.
	When in a relationship, I am always ready to apologize and forgive.	
	When in a relationship, I make sure I spend quality time alone with my partner.	
8. Religion and spirituality	I encourage my child to live a moral life.	
	I’m always available to answer my child’s spiritual questions.	
	I support my child’s spiritual development through special schooling or other means.	
	I participate with my child in the celebration of religious holidays or other religious or spiritual events.	
	I set a good example for my child by regularly participating in spiritual or religious activities.	Model appropriate desired behavior.
	I always try to support my child’s participation in legitimate spiritual or religious activities.	
	I am open about sharing my religious and spiritual beliefs with my child.	
	I always encourage my child to respect the religious beliefs of others.	
	I encourage my child to socialize with people who have spiritual or religious beliefs.	
9. Safety	I encourage my child to stay away from friends who may be a threat to his or her safety.	Monitor children’s activities and facilitate their contact with peers and adults.
	I make sure that dangerous items and substances in my home are not accessible to my child.	Monitor children’s activities and facilitate their contact with peers and adults.
	I make rules for my child that are appropriate to his or her abilities and maturity level to try to keep him or her safe.	
	I always try to teach my child how to be safe when engaging in potentially unsafe activities.	
	I keep safety items, such as a fire extinguisher and a first-aid kit, in my home.	
	I always make sure my child has appropriate supervision when I am not available.	Monitor children’s activities and facilitate their contact with peers and adults.
	I encourage my child to wear protective gear when participating in potentially dangerous activities.	Monitor children’s activities and facilitate their contact with peers and adults.
	When my child talks to me about health-related issues, problems with friends or school, or other sensitive matters, I try to be supportive and not to judge.	
	I have taught my child ways to handle emergencies, such as fires or accidents in the home, appropriate to his or her abilities and maturity level.	
10. Stress management	I plan ahead to avoid last-minute stress.	Manage personal stress.
	I’m good at putting a positive spin on things.	
	I always try to find ways to reduce sources of stress for myself and my child.	Manage personal stress.
	I often find myself overwhelmed by stress. (reversed scored)	Manage personal stress.
	I teach my child constructive ways of reducing and managing stress.	
	I occasionally take time away from my child to recharge.	
	I’m good at prioritizing.	
	I try to avoid self-destructive ways of dealing with stress.	Manage personal stress.
	I regularly practice relaxation techniques such as meditation, breathing, or imagery exercises.	Manage personal stress.

† NEPEM practices that are not represented in EPCI: 1. Ask for and accept support from others when needed. 2. Recognize one’s own personal and parenting strengths. 3. Have a sense of purpose in setting child-rearing goals. 4. Observe and understand one’s children and their development. 5. Recognize how children influence and respond to what happens around them. 6. Convey fundamental values underlying basic human decency. 7. Celebrate life with one’s children. 8. Help children feel connected to family history and cultural heritage. 9. Teach children about themselves, others, and the world around them. 10. Stimulate curiosity, imagination, and the search for knowledge. 11. Create beneficial learning conditions. 12. Help children process and manage information. 13. Find, use, and create community resources when needed to benefit one’s children and the community of children. 14. Stimulate social change to create supportive environments for children and families. 15. Build relationships with family, neighborhood, and community groups. 16. Offer support to other parents. NEPEM often provides broad guidelines in its practices, such as “Manage personal stress”; whereas, the EPCI tends to pinpoint specific behaviors. In the stress-management category (no. 10), for example, the EPCI lists nine items, five of which could be said to be subsumed under “Manage personal stress.”

A third parent education program—the Triple P-Positive Parenting Program—has been in use in multiple countries since 1970 (see Triple, 2024), and its value has also been demonstrated empirically (Li et al., 2021; Nogueira et al., 2022; Sanders, 1999). It too can be viewed from a competencies perspective, and some of the practices it teaches also overlap with those in the present study. Triple P differs from our questionnaire and some of the other programs we have mentioned because it focuses mainly on children at risk (Li et al., 2021; cf. Spoth and Redmond, 1996). Triple P also charges for its products and services, whereas our questionnaire is a free online assessment.

### 1.2 Advantages of a competencies approach

Inspired in part by the work of David C. McClelland (1973) and others (e.g., Briscoe and Hall, 1999; Deming, 1982; Quinn et al., 1990), a competencies approach to assessment and training has long been common in the business community (e.g., Shippmann et al., 2000), the military (e.g., Zook, 2006), healthcare (e.g., Marrelli et al., 2005), education (e.g., Warn and Tranter, 2001), and other areas in which human performance is important (e.g., Epstein and Phan, 2012; Epstein et al., 2008, 2013a,b, 2016, 2022, 2024, 2025a,b).

A competencies approach is advantageous because it breaks down complex performances into a set of measurable, trainable skills (Markus et al., 2005). Because these skills are trainable, competency tests give people hope; however low someone’s current level of functioning, it can presumably be improved. Unlike instruments that measure personality traits, a competencies inventory avoids labeling people in ways that can demoralize or lead to a stereotype threat (Granello and Gibbs, 2016; Link, 1987; Miller, 2017; Steele and Aronson, 1995; Steele et al., 2002). In addition, a competencies approach emphasizes the empirical study of directly observable behavior; hypothetical constructs, which often guide inquiries in the behavioral sciences, cannot be directly observed.

The value of a competencies approach to parenting has been shown in a long-running program (since 1983) at Iowa State University developed by Spoth and Redmond (1996). This program has demonstrated the value of training both parenting and family competencies in multiple controlled studies (e.g., Becher et al., 2022; Molgaard et al., 2000; Spoth et al., 2008), focusing primarily on interventions affecting substance abuse in young adolescents. Because of that emphasis, the range of competencies has been tailored to families and young people at risk. That generally has meant focusing on a range of behavior management skills (e.g., consistent discipline, clear parental expectations, using consequences, child management practices), stress management (e.g., handling stress, emotional management skills), as well as on skills for improving the parent-child bond.

### 1.3 More recent approaches to parenting competence

In 2014, Brian Johnson and his colleagues proposed an empirically-based model of parenting competence, which they defined as the “*learned ability* to effectively rear children by performing requisite tasks using demonstrable knowledge, skills or abilities, practices or behaviors, attributes, attitudes, and clusters of these components (styles) associated with positive outcomes in children” (Johnson et al., 2014, p. 94, cf. Nexford Staff, 2024). Identifying trainable parenting competencies, they argued, will help to improve both parenting practices and outcomes. The Johnson et al. (2014) definition of “competencies” is the one we will employ in the present study. Note that under this definition, competencies is a broader concept than skills—one we find to be especially useful.

Our own effort to use a competencies approach to understanding parenting began in 2006 with a review of empirical studies suggesting that particular parenting competencies were associated with good outcomes with children. Our review yielded a set of 10 relatively distinct competencies, which we delineate in the Method section below (also see Table 2 for further details and references that demonstrate empirical support for each competency).

**TABLE 2:** Ten important parenting competencies.

Competency	Sample item	References
1. Autonomy and independence *You treat your child with respect and encourage him or her to become self-sufficient and self-reliant.*	“I try to teach my child how to solve problems on his or her own.”	Benito-Gomez et al., 2020 Bronstein et al., 1996 Faber, 2002 Flouri, 2004 Grolnick, 2003 Hoeve et al., 2011 Joussemet et al., 2008 Knee and Uysal, 2011 Liu et al., 2009 Scotto Rosato and Schmitz, 2006 Soenens et al., 2007 Warash and Markstrom, 2001 Wong et al., 2002
2. Behavior management *You provide the proper balance of affection and discipline.*	“I reward my child far more often than I punish him or her.”	Ateah, 2003 Banks, 2002 Bombi et al., 2015 Breitenstein et al., 2012; Cleveland et al., 2005 Cowan et al., 2019 Dadds et al., 2003 Gordon and Cui, 2012 Lansford et al., 2012 Marchant et al., 2004 Meteyer and Perry-Jenkins, 2009 Morrissey and Gondoli, 2012 National Institute of Child Health and Human Development Early Child Care Research Network, 2005; Perepletchikova and Kazdin, 2004; Wang et al., 2015
3. Education and learning *You promote and model learning for your child.*	“I encourage my child to read.”	Dotterer et al., 2012 Gordon and Cui, 2012 Herbers et al., 2011 Irwin and Elley, 2011 Ma et al., 2016 McWayne et al., 2004; Myrberg and Rosén, 2008; Schlee et al., 2009 Snowden and Conway, 1996; Sturge-Apple et al., 2008 Suizzo et al., 2012 Warash and Markstrom, 2001
4. Healthy lifestyle *You model and provide a healthy lifestyle and good health habits for your child.*	“I try to serve healthful meals at home.”	Archibald et al., 2002 Birch, and Davison, 2001; Cleveland et al., 2005 Gerards and Kremers, 2015 Haugstvedt et al., 2011 Morgan et al., 2011 Patrick and Nicklas, 2005; Stenhammar et al., 2007 West et al., 2010
5. Life skills *You provide for the child, have a steady income, and plan for the future.*	“I typically have a steady income sufficient to cover my expenses.”	Cumella et al., 1998 Herbers et al., 2011 Katz et al., 2011 Kolos et al., 2009 Mann et al., 2004 McMorris et al., 2002 Nash, 2008 Rafferty et al., 2004
6. Love and affection *You support and accept the child and express love and affection for him or her. You spend quality one-on-one time with him or her.*	“No matter how busy I am, I try to spend quality time with my child.”	Cline and Fay, 2014 Daly, 1996 Faber, 2002 Hu and Cai, 2008 Johnson et al., 2006 Knafo and Plomin, 2006; Rohner et al., 2005 Schrodt et al., 2007; Vyšniauskytė-Rimkienė, 2019
7. Relationship skills *You maintain a positive relationship with your partner, spouse, or co-parent, and you model effective relationship skills with other people.*	“When in a relationship, I make sure I spend quality time alone with my partner.”	Benzies et al., 2004 Black, 2022 Choi and Becher, 2019 Favez et al., 2022 Nunes et al., 2020 Oh et al., 2021 Perez-Brena et al., 2020 Sturge-Apple et al., 2008
8. Religion and spirituality *You support your child’s spiritual or religious development. You also participate in spiritual or religious activities with your child.*	“I am open about sharing my religious and spiritual beliefs with my child.”	French et al., 2008 Godina, 2014 Hong et al., 2008 Kelley et al., 2020 Lau and Yuen, 2013 Mahoney, 2010 Simons et al., 2004 Vermeer, 2011 Weyand et al., 2013
**9. Safety** *You take precautions to protect your child, maintain awareness of the child’s activities and friends, and make the child feel safe about disclosing secrets.*	“I encourage my child to stay away from friends who may be a threat to his or her safety.”	Hummer et al., 2013 Kendrick et al., 2008 Miller et al., 2018 Morrongiello et al., 2008
**10. Stress management** *You take steps to reduce stress for yourself and your child, practice relaxation techniques, and promote positive interpretations of events. You also take a proactive approach to stress management.*	“I’m good at putting a positive spin on things.”	Benzies et al., 2004 Cabecinha-Alati et al., 2020; Lessenberry and Rehfeldt, 2004 Maggio, 2008 Putnick et al., 2008 Tharner et al., 2012 Yehuda et al., 2008

The model introduced by Johnson et al. (2014) is hierarchical; the structure of the EPCI is linear. Both are empirically-based, and there is considerable overlap in the competencies we delineated and those proposed by Johnson et al. (2014). Johnson’s sub-competency, “nurturance,” corresponds to our Love and Affection competency (Table 2). Both the Johnson model and the EPCI value the encouragement of autonomy and independence. Both also include behavior management (“behavioral guidance” in Johnson et al., 2014) as one of the competencies, emphasizing the need for discipline and positive reinforcement in encouraging positive behaviors in children.

Our review of relevant literature also suggested that building and maintaining relationships both within and outside the family is important for a child’s development; hence the inclusion of Relationship Skills in our model; similarly, Johnson et al. (2014) included “social network, social and friendship opportunity,” and “social skill instruction,” among their sub-competencies. Both models also included stress management, education, and safety and health among their competencies.

Johnson et al. (2014) presented a strong case for developing a competencies approach to understanding parenting. In the present study, we also attempt to do so in three ways. First, as noted above, we developed an instrument for measuring parenting competencies based on a review of relevant literature. Second, we used a “concurrent study” design (American Educational Research Association, 2014, p. 17) to evaluate some aspects of the reliability and validity of the testing instrument using data we obtained from a diverse sample of parents in multiple countries. Third, we used regression analyses to rank order our competencies according to how well they predicted a number of self-reported parenting outcomes.

The present study presents an analysis of data collected from the time the EPCI was first posted in 2007 through January 2026. Given the large sample of participants (*N* = 16,986), the diversity of the sample (133 countries), and the wide range of competencies evaluated on the questionnaire, we believe this analysis sheds new light on the importance of parenting competencies. The large sample size also allowed us to look at demographic differences in scores, as well as to use regression analyses to compare the relative importance of the competencies.

## 2 Materials and methods

### 2.1 Ethics statement

The federally registered Institutional Review Board (IRB) of the sponsoring institution (American Institute for Behavioral Research and Technology) approved this study (protocol number 10011) with exempt status and a waiver of the requirement for informed consent under the U.S. Department of Health and Human Services (HHS) regulations [45 CFR 46.116(d), 45 CFR 46.117(c)(2), and 45 CFR 46.111] because (a) the anonymity of participants was preserved and (b) the risk to participants was minimal. The IRB is registered with the Office for Human Research Protections (OHRP) under number IRB00009303, and the Federalwide Assurance number for the IRB is FWA00021545.

### 2.2 Participants

Data were initially cleaned as follows: If an individual completed the questionnaire more than once in a single day (detectable from the date and time recorded for each test session), only the first set of results in which he or she answered at least half the questions was retained. No participants reported English fluency levels under 6 (on a scale from 1 to 10). Individuals who did not identify themselves as parents were removed from the data.

We started the cleaning process with a total of 17,912 participants. After cleaning, our dataset consisted of 16,986 online participants who had completed the EPCI between April 15, 2007 and January 7, 2026. A detailed breakdown of the sample by demographic characteristics is given in Table 3.

**TABLE 3:** Demographic comparisons.

Demographic category	*n* (%)	Mean total score (*SD*)	Test statistic	*p*	Effect size
Age (M = 37.4, SD = 9.9, median = 37)					
14–37^†^	9,068 (53.4)	80.4 (13.0)	*U* = 35,077, 475.50	0.176 NS	*r* = 0.01
38–84	7,831 (46.1)	80.4 (12.3)	ρ = −0.02	0.043	−
Number of children (M = 2.3, SD = 1.4, median = 2)					
1–2	11,370 (66.9)	80.4 (12.3)	*U* = 30,690, 290.00	0.007	*r* = 0.02
3–10	5,539 (32.6)	80.4 (13.5)	ρ = 0.02	0.045 NS	−
Gender					
Female	12,817 (75.5)	80.9 (12.2)	*H* = 68.77	< 0.001	*E^2^_*R*_* = 0.004
Male	4,110 (24.2)	79.0 (14.0)			
Other	54 (0.3)	74.6 (16.9)			
Female vs. male					
Female	12,817 (75.5)	80.9 (12.2)	*U* = 24,208, 605.50	< 0.001	*r* = 0.06
Male	4,110 (24.2)	79.0 (14.0)			
Race/ethnicity					
American Indian	157 (0.9)	81.8 (11.1)	*H* = 78.99	< 0.001	*E^2^_*R*_* = 0.005
Asian	760 (4.5)	77.7 (15.0)			
Black	1,510 (8.9)	81.4 (14.9)			
Hispanic	928 (5.5)	79.4 (14.8)			
Other	599 (3.5)	79.2 (14.3)			
White	12,860 (75.7)	80.5 (12.0)			
Asian vs. Non-Asian					
Asian	760 (4.5)	77.7 (15.0)	*U* = 5,480, 080.50	< 0.001	*r* = 0.04
Non-Asian	16,053 (94.5)	80.5 (12.5)			
Level of education					
None	940 (5.5)	78.6 (16.6)	*H* = 11.84	0.037	*E^2^_*R*_* = 0.001
High school	4,542 (26.7)	79.6 (14.7)			
Associate	1,542 (9.1)	80.6 (12.2)			
Bachelor’s	5,134 (30.2)	80.8 (11.6)			
Master’s	3,706 (21.8)	80.8 (10.8)			
Doctorate	985 (5.8)	82.0 (10.8)			
Sexual orientation					
Straight	15,444 (90.9)	80.5 (12.6)	*H* = 44.41	<0.001	*E^2^_*R*_* = 0.003
Bisexual	602 (3.5)	78.4 (12.8)			
Gay or lesbian	193 (1.1)	79.9 (14.5)			
Other	191 (1.1)	77.0 (13.7)			
Straight vs. non-straight					
Straight	15,444 (90.9)	80.5 (12.6)	*U* = 6,801, 914.00	<0.001	*r* = 0.04
Non-straight	986 (5.7)	78.4 (13.4)			
Straight vs. gay/lesbian					
Straight	15,444 (90.9)	80.5 (12.6)	*U* = 1,466, 514.50	0.702 NS	*r* = 0.00
Gay or lesbian	193 (1.1)	79.9 (14.5)			
Country					
United States	9,050 (53.3)	80.7 (12.8)	*U* = 19,392, 925.00	<0.001	*r* = 0.08
Other	4,757 (28.0)	79.4 (11.8)			
English fluency	−	−	ρ = 0.15	<0.001
Marital status: currently married					
Yes	10,518 (61.9)	80.6 (12.0)	*U* = 32,910, 718.00	0.309 NS	*r* = 0.01
No	6,317 (37.2)	80.1 (13.8)			
Marital history					
Ever been married					
Yes	13,135 (77.3)	80.5 (12.3)	*U* = 24,125, 214.50	0.536 NS	*r* = 0.00
No	3,698 (21.8)	79.9 (14.3)			
Ever been divorced					
Yes	4,439 (26.1)	80.8 (12.9)	*U* = 26,358, 733.50	<0.001	*r* = 0.03
No	12,371 (72.8)	80.4 (12.7)			

Significance tests and *p*-values compare mean total scores.

† IRB approval had been received for people as young as 12 given that the Flesch-Kincaid reading level of the EPCI is 7.2. Only 10 participants were age 14, and, in all, just 147 participants were under age 18–0.9% of the data set.

### 2.3 Study design and questionnaire construction

As noted earlier, to determine which competencies might contribute to parenting success, we first reviewed the relevant literature, seeking empirical papers that found associations between specific parenting practices, skills, abilities, and traits, and desirable parenting outcomes (cf. Moher et al., 2009). Our review at that time yielded a set of 10 relatively distinct competencies, which we labeled as follows: (1) Autonomy and Independence, (2) Behavior Management, (3) Education and Learning, (4) Healthy Lifestyle, (5) Life Skills, (6) Love and Affection, (7) Relationship Skills, (8) Religion and Spirituality, (9) Safety, and (10) Stress Management. Definitions and relevant references, which have been updated since the questionnaire was first posted in 2007, are given in Table 2.

Employing procedures developed by the first author in the development of other online tests (e.g., Epstein, 2000a,b; Epstein and Muzzatti, 2011; Epstein and Rogers, 2001; Epstein and Phan, 2012; Epstein et al., 2008, 2013a,b, 2016, 2017, 2022, 2023), we then generated test items derived from the findings of relevant studies. All test items used an agree/disagree format and a 5-point Likert scale.

The questionnaire was made publicly available online.^1^ Over time, links to the questionnaire were posted at other websites, a process over which the authors had no control. The following sections detail each section of the EPCI and are ordered the same way that questionnaire content was presented to participants during their online experience.

#### 2.3.1 Demographics

Participants were first presented with brief instructions informing them, among other things, that there were no right or wrong answers to the questions. They were then asked some basic demographic questions, including a question about race/ethnicity. We are aware that the terms “race” and “ethnicity” refer to different aspects of group differences and that the use of the term “race,” in particular, is often debated (see American Psychological Association, 2019; Causadias et al., 2018; Helms et al., 2005; Roberts et al., 2020; Yee, 2009). In our questionnaire, participants were asked to select one category under the heading “Race/Ethnicity,” and the options were American Indian, Asian, Black, Hispanic, White, and Other—categories used by the United States Census Bureau when the questionnaire was first posted. We are therefore using these categories in the present paper.

#### 2.3.2 Success criteria

Following the demographic questions, participants were asked five criterion questions, the first four of which were about outcomes associated with successful parenting, namely: *How happy have your children been? How healthy have your children been? How successful have your children been in school or work settings? How good has your relationship been with your children?* The fifth criterion question measured participants’ self-reported parenting ability: *How good of a parent do you think you are?* Answers were given on 10-point Likert scales from Low to High.

#### 2.3.3 Main questionnaire

After the five criterion questions, participants completed the 100-item questionnaire itself. The test consisted of 90 scored items (nine for each competency) plus 10 unscored “dummy” items (one for each competency), all of which were presented to participants in the same random order. Each dummy item was a close variant of one of the scored items; for example, one scored item in the Healthy Lifestyle competency was “I regularly take my child to the doctor and dentist for check-ups,” and its dummy variant was “I take my child in for medical check-ups on a regular basis.” The dummy items (1, 4, 30, 32, 36, 46, 56, 63, 69, and 70) were not scored. Dummy items were included to allow us to compute an “internal consistency score” (ICS) for each participant (see Supplementary Text 1 to see how we compute ICS).

Normally, the ICS allows us to identify participants who are responding inconsistently to questionnaire items, perhaps because of a lack of attention or language difficulties. When the ICS is below a certain value, participants can be asked to retake the questionnaire or can be eliminated from an analysis of data. In the present study, we did not ask any participants to retake the questionnaire, and we did not eliminate any participants from the analysis. We had no need to remove participants with low ICSs because even people with the lowest ICS values we found generated item scores with Cronbach alpha values exceeding 0.90. The usual minimum alpha value considered acceptable in evaluation studies is 0.70 (see alpha values in Supplementary Table 1; Bland and Altman, 1997; Cortina, 1993).

After completing the 100-item questionnaire itself, participants were given their overall score as well as scores on the 10 subscales and detailed explanations about the nature of each competency.

### 2.4 Independent ratings of content

Before the questionnaire was posted online, a recently retired professor of child and family development, assisted by the director of a parenting training program (see Acknowledgments) conducted a double-blind procedure on our behalf to evaluate the questionnaire content. She recruited 11 parenting experts in at least seven U.S. states; four of the 11 experts did not provide a location, but all were in the U.S. Five were licensed clinicians. On 10-point scales, each evaluator rated both the importance of the 10 competencies and the appropriateness of each of the 9 scored items within each competency (Table 4).

**TABLE 4:** Ratings obtained from experts in double-blind procedure, ranked highest to lowest.

Competency	Mean rating of importance of competency (*SD*)	Mean rating of appropriateness for all scored items (*SD*)	Items	Mean rating of appropriateness for each scored item (*SD*)
1. Love and affection	9.6 (0.9)	9.2 (1.1)		
			i33	9.7 (0.6)
			i99	9.6 (0.9)
			i100	9.5 (0.8)
			i60	9.5 (1.0)
			i35	9.4 (1.1)
			i95	9.1 (1.2)
			i96	9.0 (1.8)
			i10	8.7 (1.6)
			i6	8.6 (2.0)
2. Relationship skills	9.2 (1.0)	8.0 (1.4)		
			i58	9.0 (1.6)
			i3	8.8 (1.6)
			i25	8.5 (1.5)
			i62	7.8 (3.2)
			i91	7.2 (3.6)
			i71	7.0 (3.5)
			i64	6.9 (2.8)
			i40	6.8 (2.9)
			i26	6.6 (2.9)
3. Autonomy and independence	9.1 (1.1)	8.6 (1.3)		
			i68	9.9 (0.3)
			i79	9.5 (1.3)
			i29	9.1 (1.2)
			i44	8.9 (1.9)
			i49	8.8 (1.1)
			i89	8.5 (2.1)
			i97	8.3 (2.3)
			i92	8.1 (2.6)
			i8	6.7 (3.0)
4. Education and learning	9.1 (1.3)	8.6 (1.3)		
			i21	9.9 (0.3)
			i88	9.2 (1.3)
			i76	9.1 (1.1)
			i17	8.9 (1.6)
			i67	8.7 (1.3)
			i90	8.5 (2.4)
			i74	8.4 (1.3)
			i66	7.4 (2.8)
			i11	7.3 (2.2)
5. Healthy lifestyle	9.0 (1.4)	9.2 (1.0)		
			i19	9.5 (0.7)
			i15	9.5 (1.2)
			i52	9.4 (0.8)
			i24	9.4 (0.9)
			i80	9.3 (0.9)
			i16	9.1 (1.0)
			i85	9.0 (1.7)
			i28	8.8 (1.5)
			i98	8.5 (1.9)
6. Safety	8.9 (1.2)	9.4 (1.0)		
			i77	9.7 (0.6)
			i82	9.7 (0.6)
			i48	9.6 (0.7)
			i75	9.5 (0.8)
			i50	9.5 (1.0)
			i73	9.5 (1.0)
			i51	9.2 (1.4)
			i81	9.1 (1.7)
			i23	9.0 (1.3)
7. Life skills	8.3 (1.2)	8.3 (1.2)		
			i18	9.4 (1.3)
			i57	8.3 (1.6)
			i9	8.2 (2.8)
			i45	7.9 (2.7)
			i53	7.5 (2.7)
			i43	7.5 (2.8)
			i31	7.5 (3.4)
			i61	7.4 (2.6)
			i41	6.4 (3.1)
8. Stress management	8.2 (1.0)	8.1 (1.4)		
			i65	8.9 (0.8)
			i38	8.3 (2.5)
			i86	8.1 (1.6)
			i83	7.8 (3.0)
			i2	7.7 (2.7)
			i87	7.5 (3.2)
			i27	7.2 (3.0)
			i47	6.9 (3.8)
			i93	5.5 (3.9)
9. Behavior management	7.0 (2.9)	6.6 (1.8)		
			i42	8.6 (2.7)
			i72	7.5 (3.2)
			i84	7.3 (2.9)
			i59	7.3 (3.0)
			i14	7.2 (2.8)
			i78	6.2 (3.7)
			i54	6.0 (3.6)
			i20	4.7 (3.1)
			i94	4.6 (3.6)
10. Religion and spirituality	6.5 (3.2)	7.3 (1.6)		
			i5	9.3 (1.8)
			i39	9.1 (1.4)
			i37	7.6 (2.9)
			i7	7.5 (3.0)
			i13	6.6 (3.7)
			i34	5.8 (3.7)
			i22	5.5 (3.5)
			i12	5.3 (3.9)
			i55	5.2 (3.6)
All competencies	8.5 (1.2)	8.3 (1.1)		

### 2.5 Statistical analysis plan

Because the EPCI is a new measurement instrument, we sought to evaluate some aspects of its reliability and validity. We estimated the EPCI’s internal consistency by using Cronbach’s Alpha and the Guttman Split-Half Test. Because the EPCI is an internet-based questionnaire, and because our IRB required that the participants take the EPCI anonymously, we did not have a way of evaluating test-retest reliability.

Our investigation employed a concurrent study design that provided convergent validity evidence with related measures, following the most recent guidelines of Standards for Educational and Psychological Testing, jointly published by the American Educational Research Association (AERA), the APA, and the National Council on Measurement in Education (American Educational Research Association, 2014). Specifically, as a preliminary measure of validity, we sought to measure the strength of the relationships between our questionnaire scores and the scores on five criterion questions. This design is called “concurrent” because we obtained test scores and criterion measures at the same time, a strategy that avoids possible temporal confounds. Results from studies employing this design are considered especially robust when the pattern of relationships between questionnaire scores and criterion measures proves to be consistent across different demographic groups (see Results). We also compared mean scores (using a Mann-Whitney U) between participants who reported having parenting training and those who did not. Presumably, if the EPCI is a valid measure of parenting competence, we should find a significant difference between the mean scores of people with training and those without.

Because questions on the EPCI use an ordinal scale, non-parametric statistics, such as Spearman’s ρ (for correlations), the Mann-Whitney U (for comparing two means), and the Kruskal-Wallis H (for comparing three or more means), are used throughout this report. Means and standard deviations are reported for comparison purposes, although the appropriateness of their use with ordinal data has long been debated (e.g., Lord, 1953; Townsend and Ashby, 1984). Questionnaire scores are always given as a percentage of the maximum possible raw score.

We used linear regression to assess which of our 10 parenting competencies best predicted answers to our five criterion questions. We also conducted demographic analyses by comparing mean scores of different demographic groups within the same demographic category. Because the data were collected over a period of 19 years, we also looked for changes in our data over time.

When reporting statistical significance, we followed the guidelines of the 7th edition of the *Publication Manual of the American Psychological Association* (American Psychological Association, 2020); we reported the actual *p-*value when that value was equal to or larger than 0.001; when the value was below 0.001, we reported *p* <0.001.

## 3 Results

### 3.1 Reliability and validity

Both the Cronbach’s alpha (0.97) and Guttman split-half (0.95) measures suggested that the EPCI has high internal-consistency reliability. Content validity was assessed using the double-blind procedure described above (see Table 4). Love and Affection was rated the most important competency by the independent evaluators (*M* = 9.6, *SD* = 0.9), and Religion and Spirituality was rated the least important competency (*M* = 6.5, *SD* = 3.2). The mean importance rating for all competencies was 8.5 (*SD* = 1.2), and the mean appropriateness rating for the items was 8.3 (*SD* = 1.1).

Again, because our IRB required us to maintain the anonymity of our participants, we did not have a way to contact our participants to ask them to complete an additional questionnaire to compare our questionnaire scores to as a measure of convergent validity. Following AERA guidelines, validity evidence was obtained by calculating the correlations between total scores and the answers to our five criterion questions. All five of our criterion measures were positively correlated with total scores, with the correlation for quality of the relationship with the children especially high (ρ = 0.45, *p* < 0.001) (Table 5).

**TABLE 5:** Validity evidence based on self-reported criterion measures.

Criterion question	Correlation with total score
Quality of relationship with children	0.45***
Self-reported parenting ability	0.43***
Happiness of children	0.40***
Health of children	0.27***
Success of children	0.30***

***p <0.001.

Two of our measures of parenting training also provide validity evidence: First, people who reported having had such training scored significantly higher on the questionnaire than people who did not [*M*_*yes*_ = 83.0 (*SD* = 11.4), *M*_*no*_ = 79.2 (13.1), *U* = 24,502, 725.50, *p* <0.001, *r* = 0.15]. Second, the number of training hours people reported was positively correlated with their total score on the questionnaire (ρ = 0.11, *p* < 0.001).

### 3.2 Regression analysis

The relative predictive value of the 10 competencies was assessed using linear regression (Tables 6, 7). Love and Affection proved to be the best predictor of two of the criterion variables: Relationship with Children, and Happiness of Children. Stress Management proved to be the best predictor of Parenting Ability. Success of Children was best predicted by the Education and Learning competency. Health of Children was best predicted by the Healthy Lifestyle competency. Safety made significant *negative* contributions in four of the regressions (standardized betas: β*_*Relationship*_* = −0.18, β*_*Happiness*_* = −0.13, β*_*Parenting*_* = −0.22, and β*_*Success*_* = −0.07) and did not make significant positive contributions in any of the regressions (Note: Using the language of prediction here in the context of regression analysis is not meant to imply causal relationships among the variables).

**TABLE 6:** Linear regressions predicting criterion variables from competency scores (best predictor standardized β).

Criterion variable															
	Relationship with children			Happiness of children			Parenting abilities			Success of children			Health of children		
Competencies	β^†^	*t*	*R* ^2^	β	*t*	*R* ^2^	β	*t*	*R* ^2^	β	*t*	*R* ^2^	β	*t*	*R* ^2^
Love and affection	0.48***	71.51	0.23	0.40***	56.09	0.16									
Stress management							0.43***	61.35	0.18						
Education and learning										0.28***	36.94	0.08			
Healthy lifestyle													0.26***	34.8	0.07

*** p <0.001.

† β Standardized beta weight from single-component model in a stepwise regression. No causal relationships are implied.

**TABLE 7:** Regression results showing how well the 10 competencies predicted answers to the criterion questions.

Competency	Happiness			Relationship Quality			Parenting Ability			Health			Success		
	β^†^	*t*	*R* ^2^	β^†^	*t*	*R* ^2^	β^†^	*t*	*R* ^2^	β^†^	*t*	*R* ^2^	β^†^	*t*	*R* ^2^
Love and affection	0.23***	16.49	0.20	0.37***	28.31	0.27	0.10***	7.33		0.07***	5.50		NA	NA	
Autonomy and independence	0.09***	6.60		0.12***	7.92		0.05***	3.20		0.04*	2.53		0.09***	7.15	
Stress management skills	0.11***	9.65		0.16***	15.81		0.32***	28.59	0.21	0.04***	3.29		0.05***	4.06	
Relationship skills	0.10***	9.33		0.06***	6.45		0.05***	5.01		0.05***	4.67		0.04***	3.89	
Behavior management	0.07***	5.76		0.08***	6.42		0.07***	6.05		NA	NA		NA	NA	
Life skills	0.03*	2.46		NA	NA		0.10***	9.70		0.06***	5.16		0.05***	4.23	
Healthy lifestyle	0.04***	3.40		NA	NA		0.04***	3.73		0.10***	8.09	0.09	0.04**	3.02	
Religion	−0.02*	−2.04		−0.02**	−2.81		NA	NA		NA	NA		NA	NA	
Safety	−0.13***	−9.92		−0.18***	−14.65		−0.22***	−17.30		NA	NA		−0.07	−5.20	
Education	−0.03*	−2.43		−0.03**	−2.78		−0.06***	−4.97		NA	NA		0.15***	11.00	0.09

Results are shown for one-component models in stepwise regressions, one for each of the five criterion questions.

† β Standardized beta weight from single-component model in a stepwise regression. *p < 0.05, **p <0.01, ***p <0.001.

Table 8 compares the ranking of the 10 competencies according to questionnaire scores, expert scores, and regression betas. It suggests that there is a mismatch in some cases between the level of competence parents actually have (Mean Questionnaire Scores) and the value of those competencies as determined by experts and/or regression analyses. The most dramatic mismatch is for the Safety competency, which parents generally excel in but which may have little value and might even be harmful (Ganaprakasam et al., 2023; Gray et al., 2023; Perry et al., 2018; Salmin et al., 2021; Suarex-Morales and Harris, 2023; Zhang and Wang, 2024). Note that we did not ask parents to rate their competencies in the 10 categories measured by the questionnaire, so we have no way of comparing perceived competence with the questionnaire scores, expert scores, or regression betas.

**TABLE 8:** Comparing rankings of competencies based on questionnaire scores, expert scores and regression analyses.

Competencies	Mean questionnaire scores	Parenting experts	Regressions^†^
Safety	1	6	−
Love and affection	2	1	1
Education and learning	3	4	3
Healthy lifestyle	4	5	4
Autonomy and independence	5	3	−
Life skills	6	7	−
Behavior management	7	9	−
Religion and spirituality	8	10	−
Relationship skills	9	2	−
Stress management	10	8	2

For each of the three columns, 1 indicates the highest rank and 10 the lowest. The first column shows the ranking of the questionnaire scores based on the mean score for each of the 10 competencies. Column 2 shows the ranking of the 10 competencies based on the mean scores given by our panel of parenting experts. And column 3 shows the ranking of the competencies based on how well they predicted answers to our four criterion questions (based on regression analysis; see Table 6).

† Only four of the 10 competencies ranked highly enough for comparison purposes. See Table 7 for beta and *R*^2^ values.

### 3.3 Demographic analyses

Demographic effects are shown in detail in Tables 3, 9. Here is a summary of these findings: First, getting older was not associated with higher questionnaire scores (*U* = 35,077, 475.50, *p* = 0.176 NS, *r* = 0.01). Second, having three or more children was associated with higher scores (*U* = 30,690, 290.00, *p* = 0.007, *r* = 0.02). Third, participants from the U.S. outscored people in other countries, but the effect size was small (*U* = 19,392, 925.00, *p* < 0.001, *r* = 0.08). This difference might have occurred because participants from the U.S. reported a higher mean English fluency than people outside the U.S. The correlation between fluency level and total scores on the questionnaire was 0.15 (*p* < 0.001). Fourth, we found an effect for level of education (*H* = 11.84, *p* = 0.037, *E^2^_*R*_* = 0.001), but higher levels of education were not consistently associated with higher questionnaire scores.

**TABLE 9:** Self-reported mean parenting outcomes (criterion measures).

Demographic category		Happiness of children *(SD)*	Health of children *(SD)*	Success of children *(SD)*	Relationship with children *(SD)*	Parenting ability *(SD)*
Gender						
	Male	8.3 (1.4)	8.8 (1.3)	8.3 (1.6)	8.4 (1.6)	7.8 (1.7)
	Female	8.2 (1.5)	8.8 (1.3)	8.3 (1.7)	8.5 (1.5)	7.6 (1.7)
	Other	7.4 (2.2)	8.4 (2.1)	8.1 (2.2)	8.0 (2.3)	7.2 (2.4)
	*U* (Male vs. Female)	25,441,591.00*	26,069,519.00 NS	25,550,615.00 NS	25,268,112.50*	24,154,750.00***
	*r*	0.02	0.00	0.00	0.02	0.05
Sexual orientation						
	Straight	8.2 (1.5)	8.8 (1.3)	8.3 (1.6)	8.5 (1.5)	7.7 (1.7)
	Gay or Lesbian	8.0 (1.8)	8.7 (1.7)	8.1 (1.9)	8.3 (1.8)	7.6 (1.9)
	*U*	1358480.00 NS	1435732.50 NS	1367599.00 NS	1382761.00 NS	1455741.50 NS
	*r*	0.02	0.00	0.01	0.01	0.00
Married ever						
	Yes	8.2 (1.4)	8.8 (1.3)	8.4 (1.6)	8.4 (1.5)	7.6 (1.6)
	No	8.4 (1.6)	8.8 (1.4)	8.2 (1.9)	8.5 (1.7)	7.8 (1.9)
	*U*	21,476,712.00***	22,904,091.00***	23,757,342.50 NS	21,667,174.00***	22,002,614.00***
	*r*	0.08	0.03	0.00	0.07	0.06
Divorced ever						
	Yes	7.9 (1.6)	8.8 (1.4)	8.1 (1.7)	8.3 (1.7)	7.6 (1.7)
	No	8.3 (1.4)	8.8 (1.3)	8.4 (1.6)	8.5 (1.5)	7.7 (1.7)
	*U*	22,560,117.50***	26,669,420.50*	24,284,328.00***	24,986,725.50***	26,531,653.50 NS
	*r*	0.13	0.02	0.07	0.06	0.01
All participants		8.2 (1.5)	8.8 (1.3)	8.3 (1.7)	8.5 (1.6)	7.6 (1.7)

SD is shown in parentheses. The Mann-Whitney U was used to compare means. *p <0.05, ***p <0.001.

Fifth, we found an effect for race/ethnicity, with Asian parents scoring lower than non-Asian parents (*U* = 5,480,080.50, *p* <0.001, *r* = 0.04), perhaps because of the ways in which some Asian cultures differ from non-Asian cultures (see Discussion). Once again, the effect size here was small; we acknowledge that in a large-*N* study like the present one, a highly significant *p-*value is not necessarily indicative of a meaningful difference (Krueger and Heck, 2019). Sixth, we found an effect for sexual orientation, with self-labeled straights scoring higher than participants who did not label themselves as straight (*U* = 6,801,914.00, *p* <0.001, *r* = 0.04).

Seventh, we found an overall effect for gender (males vs. females vs. other) (*H* = 68.77, *p* <0.001, *E^2^_*R*_* = 0.004), with females outscoring males in a pairwise comparison (*U* = 24,208,605.50, *p* <0.001, *r* = 0.06). Eighth, in response to our question about self-perceived parenting ability, males rated their ability higher than females did (*U* = 24,154, 750.00, *p* <0.001, *r* = 0.05), but the effect size was small. Ninth, females reported having a better relationship with their children than males reported (*U* = 25,268, 112.50, *p* <0.01, *r* = 0.02). And tenth, although one might expect that people who had married were more competent as parents than people who had never married, we found no clear relationship between marital history and parenting ability (*U* = 24,125, 214.50, *p* = 0.536 NS, *r* = 0.00).

### 3.4 Changes over time

Although mean total scores on the questionnaire changed significantly over time, we also found that key demographic characteristics changed significantly from year to year (Table 10), presumably because of our inability to control which websites linked to our questionnaire. Dividing the sample into two segments of approximately equal size by date (before and after December 27, 2012, *n*_*before*_ = 8,493, *n*_*after*_ = 8,492), we found a significant difference in mean total scores for these two time periods, but the absolute difference in scores was small (0.5 percentage points), and so was the effect size [*M*_*before*_ = 80.6 (10.5), *M*_*after*_ = 80.1 (14.6), *U* = 34,427, 480.00, *p* < 0.001, *r* = 0.04]. This suggests that our overall findings are generally applicable over most or all of the long period of data collection.

**TABLE 10:** Mean percentage scores and demographic characteristics by year.

	Year data were collected																		Significance test^†^
Competencies	2008	2009	2010	2011	2012	2013	2014	2015	2016	2017	2018	2019	2020	2021	2022	2023	2024	2025	
Autonomy and independence	81.9 (14.8)	83.0 (14.7)	83.4 (13.2)	83.0 (12.5)	83.3 (13.5)	82.0 (14.9)	81.1 (16.7)	81.2 (15.5)	81.8 (16.0)	82.9 (15.4)	84.4 (14.8)	83.3 (15.7)	82.8 (16.5)	84.1 (14.9)	84.7 (14.8)	83.7 (15.2)	86.3 (12.9)	56.9 (39.0)	152.56***
Behavior management	77.5 (15.9)	78.9 (16.0)	79.1 (14.6)	77.8 (14.3)	78.4 (15.7)	77.5 (16.4)	77.1 (17.7)	76.8 (17.4)	77.2 (18.1)	79.3 (17.0)	80.4 (16.2)	80.3 (16.8)	78.4 (19.0)	81.1 (16.4)	81.1 (17.0)	81.9 (17.1)	82.6 (15.9)	56.6 (37.1)	212.13***
Education and learning	84.8 (13.6)	86.1 (14.0)	86.9 (11.7)	85.8 (12.0)	86.0 (12.8)	84.6 (14.6)	83.5 (15.6)	84.2 (14.7)	83.5 (15.8)	84.4 (14.8)	85.2 (13.6)	84.6 (15.9)	82.6 (16.8)	84.4 (15.0)	84.4 (15.5)	85.1 (14.4)	86.7 (13.1)	56.1 (41.1)	140.89***
Healthy lifestyle	81.7 (14.1)	84.2 (13.7)	84.2 (13.1)	84.6 (12.5)	83.6 (13.4)	81.2 (16.1)	81.5 (15.7)	81.5 (16.0)	81.5 (15.9)	81.9 (15.9)	83.6 (15.0)	82.9 (15.5)	81.9 (17.7)	82.3 (16.3)	84.4 (15.5)	85.2 (12.8)	85.7 (13.1)	57.1 (38.9)	145.45***
Life skills	80.7 (15.0)	82.6 (14.4)	83.6 (12.2)	83.8 (11.9)	83.5 (12.8)	81.8 (14.7)	81.7 (15.4)	82.1 (14.8)	81.7 (15.6)	82.2 (15.4)	82.8 (14.1)	83.5 (15.2)	82.1 (16.2)	84.2 (14.8)	82.9 (15.9)	83.7 (14.0)	84.2 (13.8)	55.5 (37.4)	135.52***
Love and affection	86.9 (12.9)	87.8 (13.2)	88.3 (12.1)	86.2 (12.5)	88.5 (12.8)	87.1 (14.7)	85.8 (16.3)	86.0 (15.2)	86.0 (16.2)	88.8 (14.3)	88.7 (14.0)	88.3 (15.4)	86.4 (17.7)	88.4 (14.8)	88.9 (15.2)	89.8 (14.1)	91.2 (12.7)	57.4 (43.3)	359.58***
Relationship skills	70.3 (12.9)	72.1 (12.2)	71.6 (12.2)	71.7 (11.7)	71.2 (12.9)	71.1 (13.8)	70.5 (13.4)	70.4 (13.7)	71.4 (13.8)	72.1 (13.4)	72.5 (12.6)	72.6 (13.3)	72.0 (14.5)	72.5 (13.6)	74.7 (15.6)	78.4 (17.3)	79.9 (16.3)	55.8 (34.7)	125.91***
Religion and spirituality	78.7 (17.4)	79.5 (17.9)	80.1 (17.5)	71.2 (19.4)	77.1 (19.2)	78.0 (18.7)	77.3 (18.9)	79.5 (18.6)	77.3 (19.6)	80.2 (17.6)	81.1 (17.9)	79.7 (18.6)	78.5 (19.6)	78.5 (18.1)	79.9 (18.3)	81.0 (17.6)	79.4 (18.9)	56.2 (36.1)	567.06***
Safety	87.5 (12.6)	88.7 (12.6)	89.3 (11.2)	87.7 (11.5)	88.3 (12.1)	87.5 (13.1)	86.5 (14.7)	87.2 (14.6)	87.2 (14.4)	88.5 (14.0)	89.1 (13.3)	88.5 (15.0)	86.7 (15.5)	88.4 (13.8)	88.0 (14.0)	88.6 (13.7)	89.9 (14.0)	57.1 (42.5)	177.30***
Stress management	68.0 (17.5)	69.3 (17.8)	68.4 (16.6)	69.1 (15.1)	68.7 (16.6)	67.9 (17.2)	68.1 (18.0)	67.8 (18.1)	68.2 (18.9)	70.2 (17.3)	71.2 (17.5)	70.7 (18.3)	70.5 (19.0)	71.4 (17.9)	72.1 (17.4)	73.2 (15.7)	75.0 (15.4)	53.7 (29.9)	184.70***
Total	79.8 (11.6)	81.2 (11.7)	81.5 (10.2)	80.1 (10.0)	81.0 (10.9)	80.1 (12.3)	79.3 (13.4)	79.7 (12.9)	79.6 (13.7)	81.0 (12.8)	81.9 (12.0)	81.4 (13.3)	80.2 (14.5)	81.5 (13.2)	82.1 (13.5)	83.1 (12.3)	84.1 (12.0)	56.2 (37.0)	206.65***
Demographics																			
Mean age	38.2 (9.8)	38.7 (9.8)	39.2 (9.5)	38.9 (9.6)	38.0 (9.5)	37.5 (9.7)	35.9 (10.1)	36.1 (9.3)	36.7 (10.1)	35.1 (9.7)	35.2 (10.3)	35.9 (10.0)	36.1 (10.3)	34.8 (8.7)	36.6 (10.4)	39.1 (11.5)	36.7 (12.0)	35.1 (12.1)	23.1***
Percentage females	78.4	77.7	85.4	70.8	81.6	82.2	74.4	78.6	77.9	74.9	71.0	72.0	68.5	74.7	63.8	62.6	54.3	59.7	389.9***
Percentage whites	72.4	76.3	79.0	89.1	80.1	77.7	69.3	72.6	71.5	70.0	66.7	70.6	68.4	66.9	71.4	67.0	59.8	52.9	126.9***

Statistics are omitted for 2007 and 2026 because complete data were not available for those years. *SD* is shown in parentheses.

† A Kruskal-Wallis *H* test was used to compare annual mean test scores. A test was used to compare annual percentages of females and whites (the largest groups in the gender and race/ethnicity demographic categories, respectively). A one-way ANOVA was used to compare annual mean ages.

***p <0.001.

## 4 Discussion

### 4.1 Major findings

The present study suggests that the EPCI has value in measuring a wide range of competencies that are associated with good parenting outcomes. It also confirms, with data collected from a highly diverse group of people, the value that a competencies approach has to understanding and improving parenting practices, skills, and attitudes.

For validation purposes, our study relied on guidelines from the most recent edition of *Standards for Educational and Psychological Testing* (American Educational Research Association, 2014) for employing a “concurrent study design,” that is, one in which the strength of association between test scores and the answers to various criterion questions is measured; this measure is often called “criterion-related validity.” In the present study, all five of those associations were positive and significant at the 0.001 level, and the mean test score was an especially good predictor of the quality of the relationship parents reported having with their children. We also assessed content validity by conducting a double-blind assessment of our questionnaire’s content, with generally encouraging results (Table 4). That said, because this was an internet study in which we were required to preserve the anonymity of the participants, we could not assess other traditional forms of test validity, such as concurrent validity or predictive validity (Anastasi and Urbina, 2016); accordingly, we consider the validity evidence presented in this report to be preliminary.

Our regression analyses confirmed a traditional belief about parenting but also supported some new ideas. By far, the best predictor of good parenting outcomes was the Love and Affection competency, so perhaps John Lennon was right when he sang “all you need is love.” This competency was the best predictor of the two outcomes that many parents value a great deal: their children’s happiness and the quality of the relationship they have with their children (Hurst et al., 2023; Stearns, 2019). The Love competency was also the second best predictor of two of the three remaining criterion variables: children’s health and self-reported parenting ability. Love and Affection was also ranked highest by our parenting experts in our content validity study, and, fortunately, it ranked second among the competencies parents actually have; parents scored an impressive 87.0% on this competency (Table 11). This is good news, and this general finding about the importance of parents expressing love and affection to their children is also consistent with the findings of other studies (Faber, 2002; Johnson et al., 2006; Schrodt et al., 2007; Smith et al., 1994).

**TABLE 11:** Mean competency scores, ranked highest to lowest.

Competency	Mean percentage score (*SD*)	Cronbach’s alpha
1. Safety	87.7 (14.2)	0.84
2. Love and affection	87.0 (15.0)	0.87
3. Education and learning	84.8 (14.8)	0.82
4. Healthy lifestyle	82.8 (15.3)	0.81
5. Autonomy and independence	82.6 (15.2)	0.85
6. Life skills	82.6 (14.7)	0.76
7. Behavior management	78.3 (16.7)	0.83
8. Religion and spirituality	77.3 (19.2)	0.85
9. Relationship skills	71.7 (13.7)	0.66
10. Stress management	69.1 (17.4)	0.78
All competencies	80.4 (12.7)	0.97

Parents scored highest on the Safety competency (87.7%, Table 11), perhaps a reflection of the overprotectiveness that has come to characterize modern parenting, at least in the U.S. (Odenweller et al., 2014; Ungar, 2009; Valentine, 2004). Unfortunately, Safety ranked poorly in our study in several respects. It was ranked sixth by our experts (Table 8), and, as we noted earlier, it appeared to be *harmful* in four of our five regressions. Other studies have suggested why an excessive concern with safety might be harmful in parenting (Ganaprakasam et al., 2023; Gray et al., 2023; Perry et al., 2018; Salmin et al., 2021; Suarex-Morales and Harris, 2023; Zhang and Wang, 2024).

Two other competencies that fared poorly in the study also deserve some mention. Our experts ranked Religion and Spirituality lowest among the competencies; its mean rating was only 6.5 (Table 4). It was, in fact, the only competency that provoked negative written comments from our blind reviewers, two of whom recommended that we omit this competency entirely, even though we had found studies suggesting its value (Table 2). Even more surprising—especially to the first author of this study, who was the last doctoral student of behavioral psychologist B.F. Skinner—was the fate of the competency we called Behavior Management. It ranked seventh among parenting scores, ninth among our experts, and *seventh* in our regressions. Skinner would have viewed such a finding to be “puzzling.”

Our methodology allowed us to collect data from a large and diverse sample of English-speaking people around the world; the shifting demographics year after year contributed greatly to the diversity of the sample (although see the Limitations section below). There is also a growing and substantive literature suggesting that people are particularly honest when answering questions about themselves anonymously online, especially when they are being asked about sensitive issues such as parenting ability (Corrigan, 2004; Dillman et al., 2014; Durant et al., 2002; Dwight and Fiegelson, 2000; Joinson, 1999; Kaplan and Saccuzzo, 2009; Krumpal, 2013; Manderscheid et al., 2010; Ong and Weiss, 2000; Robertson et al., 2017; Trau et al., 2013; Villarroel et al., 2006; cf. Reips, 2012). Presumably, because parents were taking the EPCI out of curiosity rather than out of necessity, it seems unlikely that they were inflating their scores to impress peers or authorities.

Our demographic analyses provided support for some common beliefs about parenting but also yielded some surprises. The commonly held belief that females are better parents than men are, for example, was modestly supported by our data (cf. Abraham et al., 2014; Kinsley et al., 2006). The difference we found between the mean scores for males and females was statistically significant, but the absolute difference was small (females outscored males by 2.0%), and so was the effect size (*r* = 0.06). Men, moreover, rated their parenting ability slightly higher than women rated their own parenting ability, whereas women judged the quality of their relationship with their children to be better than the quality of the relationship men reported with their children (Table 9).

Another common belief is that women produce better parenting outcomes; in our study, however, gender appeared to make no difference in parenting outcomes (e.g., self-reported happiness, health, and success of children) (Table 9). Notably, 75.5% of our participants were female (Table 3), which suggests that female internet users have a stronger interest in parenting issues than do male internet users. In general, females are also more likely to participate in research than men (Curtin et al., 2000; Moore and Tarnai, 2002; Singer et al., 2000; Smith, 2008).

Our study also confirmed the value of parenting training (e.g., Adler-Baeder et al., 2018; Becher et al., 2022; Chacko et al., 2013; Wolford and Holtrop, 2020). As noted, participants in our study who had had such training scored significantly higher on our questionnaire than people who had not had such training, and questionnaire scores were positively correlated with the number of training hours.

Some segments of the American public have long voiced the view that gay and lesbian individuals make poor parents. Our study supports the findings of other recent studies that dispel this myth (e.g., Bos et al., 2018; Farr et al., 2010; Stacey and Biblarz, 2001). Gay and lesbian participants scored the same as straight participants on our questionnaire (Table 3), and they also produced similarly positive parenting outcomes (Table 9).

Regarding race and ethnicity, Asians scored somewhat lower than non-Asians did, perhaps because some Asian cultures encourage an authoritarian style of parenting (Chiu, 1987; Cui et al., 2021; Detzer et al., 1999; Kelley and Tseng, 1992; cf. Hwang et al., 2023; King et al., 2016; Pinquart, 2017). Foreign-born Asian mothers in the U.S. might also be subject to a high degree of stress (DeLambo et al., 2011; Nomaguchi and House, 2013), which can also affect parenting adversely (Trute and Hiebert-Murphy, 2002).

We have been in the process in recent years of expanding our analysis of parenting skills by looking more carefully at cultural differences. In 2017 and 2018, and working in collaboration with the ALIAT Association for Mental Health in Bucharest, Romania, we posted translations of the EPCI in six non-English languages (Croatian, Italian, Polish, Romanian, Spanish, and Turkish). We are currently in the process of analyzing our new data by comparing both language and country differences in scores obtained by a diverse group of people who completed the questionnaire in their native languages.

### 4.2 Limitations

The main limitations of the study are as follows: First, all five of the parenting outcomes we measured were self-reported. Second, because this was an internet study that assured people’s anonymity, we were limited in how we could collect both reliability and validity evidence, and we thus remind the reader about the preliminary nature of our validation. Third, the 10 competencies we measured should not be considered a definitive list; we were constrained by relevant literature when the questionnaire was designed. Fourth, 28.0% of the participants in the study were from 132 countries outside the U.S, and we had no country data from 18.7% of the participants. The EPCI itself was derived from studies conducted mainly in the U.S.; its applicability to countries outside the U.S. is unclear, and we made no attempt in this study to examine our data from a cultural perspective, mainly because we had relatively few people in any one country outside the U.S. Although comparative analyses of this sort could be conducted using an instrument like the EPCI, the present study did not provide enough data to attempt such analyses.

Fifth, we had no control over the nature of our sample; not only were participants self-selected, but the makeup of our sample changed over time (Table 10), presumably because links to our online questionnaire kept changing. One might speculate that an online parenting questionnaire would attract parents who were worried about their parenting skills. In fact, 60.5% of our participants scored 8 or above on self-reported parenting ability, and only 4.9% of our participants gave themselves a score below 5. We know from demographic characteristics alone that our sample was unrepresentative in some respects, and we therefore cannot say with any degree of confidence that our sample was representative of parents in general. Of our participants, 57.8% had 4-year college degrees or graduate degrees, for example; in the U.S., only 35.8% of the adult population has such degrees (United States Census Bureau, 2025). Similarly, only 24.2% of our participants were male, and the men who responded to our questionnaire might differ in non-trivial ways from the population of fathers in general.

### 4.3 Implications for practice, application, and policy

Good parenting is important for children, families, and society (Baumrind, 1967; Baumrind et al., 2010; Coleman, 1988; Johnson et al., 2006; Kawachi et al., 2008; Marchant et al., 2004). The present study provides preliminary support for the potential value of a competencies approach to parenting, very much in the spirit of the position developed by Spoth and Redmond (1996), as well as by Johnson et al. (2014), who argued that “the time is propitious to begin the discussion of what competent parenting entails” (p. 108). Because the present study also suggests the relative importance of different parenting competencies, it might prove useful both in informing the ongoing discussion about parenting competencies and for guiding future research on ways to improve programs designed to improve parenting practices.

Our findings suggest in particular that after the expression of love and affection, certain practices may have special value in parenting: managing stress, promoting learning and education, promoting autonomy and independence, learning life skills, and maintaining a constructive relationship with one’s partner, spouse, or co-parent. Additionally, our results are consistent with previous research highlighting the value of parenting training, and it suggests the potential value that parenting competencies could have in producing good parenting outcomes: the happiness, health and success of children, as well as the quality of the relationship parents have with their children. Future research should aim to measure other aspects of the EPCI’s reliability and validity, while also testing the replicability of the findings in this study. Because parenting competencies can be both measured and trained, we believe that a competencies approach to parenting may be worthy of future consideration when developing parenting programs and during related discussions surrounding public policy.

## Data Availability

The raw data supporting the conclusions of this article will be made available by the authors, without undue reservation.

## Footnotes

1
https://MyParentingSkills.com

## In memoriam

We dedicate this paper to the memory of Dorothy Hewes.
