# Supplementary material for: All you need is love: preliminary validation of a new test of 10 parenting competencies that predict good outcomes with young children
Source: Front Psychol. 2026 Aug 10;17:1796786. doi: 10.3389/fpsyg.2026.1796786 (PMC13500332; doi:10.3389/fpsyg.2026.1796786)
Supplement: Supplementary file 1 [file Supplementary_file_1.docx]

**Supplementary Material**

# Text S1

*Calculation of Internal Consistency Score (ICS)*

The formula for Cohen’s kappa coefficient is:

𝑝𝑜 − 𝑝𝑒

𝑘 =

1 − 𝑝_𝑒_

where

𝑝_𝑜_ = “the proportion of units in which the judges agreed”

𝑝_𝑒_ = “the proportion of units for which agreement is expected by chance”

𝑘 = “the proportion of chance-expected disagreements which do not occur”

The formula for the ICS is:

𝑉

𝑉

where

*V* = the total possible difference in scores (e.g. a test with a Likert scale of 1-5 with four dummy pairs would have a *V* of 16, and a test with a Likert scale of 1-5 with ten dummy pairs would have a *V* of 40)

𝑥_𝑎_ = scored test items

𝑥_𝑏_ = dummy scored items

*Reference:*

Cohen, J. A. (1960). Coefficient of agreement for nominal scales. *Education and Psychological Measurement, 20*(1), 37–46. <https://doi.org/10.1177/001316446002000104>

# Table S1

# *Cronbach’s Alphas for Groups of Participants with Progressively Larger ICSs*

| **n** | **ICS** | **Cronbach’s Alpha** |
| --- | --- | --- |
| 0 | 0.0-0.1 | N/A |
| 0 | 0.1-0.2 | N/A |
| 0 | 0.2-0.3 | N/A |
| 2 | 0.3-0.4 | 0.986 |
| 29 | 0.4-0.5 | 0.990 |
| 109 | 0.5-0.6 | 0.975 |
| 342 | 0.6-0.7 | 0.970 |
| 1,978 | 0.7-0.8 | 0.962 |
| 7,321 | 0.8-0.9 | 0.956 |
| 6,178 | 0.9-1.0 | 0.961 |
